# Co-Delivery of *Dragon’s Blood* and *Alkanna tinctoria* Extracts Using Electrospun Nanofibers: In Vitro and In Vivo Wound Healing Evaluation in Diabetic Rat Model

**DOI:** 10.3390/pharmaceutics16060704

**Published:** 2024-05-24

**Authors:** Rana Y. AlMotawa, Ghadeer Alhamid, Mohamed M. Badran, Raha Orfali, Abdullah H. Alomrani, Essam A. Tawfik, Dunia A. Alzahrani, Haya A. Alfassam, Safina Ghaffar, Amany Fathaddin, Areej Al-Taweel, Aliyah Almomen

**Affiliations:** 1 Department of Pharmacognosy, College of Pharmacy, King Saud University, Riyadh 11451, Saudi Arabia; ranayalmutawa@gmail.com (R.Y.A.); alhamid.ghadeer@outlook.com (G.A.); amaltaweel@ksu.edu.sa (A.A.-T.); 2 Department of Pharmaceutics, College of Pharmacy, King Saud University, Riyadh 11495, Saudi Arabia; 3 Nanobiotechnology Unit, College of Pharmacy, King Saud University, Riyadh 11495, Saudi Arabia; 4 Advanced Diagnostics and Therapeutics Institute, Health Sector, King Abdulaziz City for Science and Technology (KACST), Riyadh 11442, Saudi Arabia; 5 Pathology Department, College of Medicine, King Saud University, Riyadh 11495, Saudi Arabia; 6 Department of Pharmaceutical Chemistry, College of Pharmacy, King Saud University, Riyadh 11495, Saudi Arabia; aalmomen@ksu.edu.sa

**Keywords:** *Dragon’s blood*, *Alkanna tinctoria*, electrospinning, wound dressing, wound healing

## Abstract

The increasing prevalence of diabetic wounds presents a significant challenge due to the difficulty of natural healing and various obstacles. *Dragon’s blood* (DB) and *Alkanna tinctoria* (AT) are well recognized for their potent healing abilities, which include potent antibacterial and anti-inflammatory activities. In this study, electrospun nanofibers (NFs) based on polyvinyl pyrrolidone (PVP) were co-loaded with both DB and AT, aiming to magnify their efficacy as wound-dressing applications for diabetic wound healing. The evaluation of these NFs as wound dressings was conducted using a streptozotocin-induced diabetic rat model. Electrospun NFs were prepared using the electrospinning of the PVP polymer, resulting in nanofibers with consistent, smooth surfaces. The loading capacity (LC) of AT and DB into NFs was 64.1 and 70.4 µg/mg, respectively, while in the co-loaded NFs, LC was 49.6 for AT and 57.2 µg/mg for DB. In addition, X-ray diffraction (XRD) revealed that DB and AT were amorphously dispersed within the NFs. The loaded NFs showed a dissolution time of 30 s in PBS (pH 7.4), which facilitated the release of AT and DB (25–38% after 10 min), followed by a complete release achieved after 180 min. The antibacterial evaluation demonstrated that the DB-AT mixture had potent activity against *Pseudomonas aeruginosa* (*P. aeruginosa*) and *Staphylococcus aureus* (*S. aureus*). Along with that, the DB-AT NFs showed effective growth inhibition for both *P. aeruginosa* and *S. aureus* compared to the control NFs. Moreover, wound healing was evaluated in vivo in diabetic Wistar rats over 14 days. The results revealed that the DB-AT NFs improved wound healing within 14 days significantly compared to the other groups. These results highlight the potential application of the developed DB-AT NFs in wound healing management, particularly in diabetic wounds.

## 1. Introduction

Diabetes mellitus (DM) is a long-term metabolic disorder marked by high blood glucose levels, which may delay the wound-healing process [1]. More than half a billion people worldwide have been diagnosed with DM, and this number is expected to double by 2050 [2]. DM is associated with some diabetic foot complications, including foot ulcers, amputations, and gangrenes, regardless of the gender or the type of diabetes [3]. It has been demonstrated that patients with diabetes and foot ulcers have a two-fold higher mortality rate than non-diabetic groups [4]. The body tissue damage of diabetes with the loss of its integrity is characterized by acute or chronic wounds. The body has a natural healing process programmed to restore and heal damaged tissues through four main biological processes: hemostasis, inflammation, proliferation, and remodeling [4]. However, if there are issues that interfere with these healing steps, a delay or impaired wound healing could occur. 

Addressing diabetic foot problems necessitates an increase in healthcare resources, leading to more hospital admissions and surgeries [5]. Patients with diabetes often struggle with foot wounds that do not heal properly owing to the malfunction of the body’s natural repair system. An abnormal connective tissue, reduced pro-collagen synthesis, and a higher level of tissue-degrading matrix metalloproteinase are considered some examples that could affect the body’s natural repair system [6]. Moreover, oxidative stress might also contribute to diabetic complications such as neuropathy and foot ulcers [7].

Numerous approaches have been developed to improve and accelerate wound healing. These include debridement, irrigation, surgical procedures, topical treatments, and wound dressings. Currently, various techniques for the preparation of wound dressing have been developed, such as self-assembly, freeze-drying, solvent casting, and electrospinning [8]. These methods are designed to obtain materials with specific properties that can promote wound healing and improve patient outcomes. Electrospinning techniques have been used to produce nanofibers (NFs), serving as wound dressings. These NFs can protect the skin from bacterial invasion and dehydration with proper mechanical support [9]. The shape and structure of NFs are highly similar to the extracellular matrix, providing great cellular processes like adhesion, migration, proliferation, and angiogenesis [10]. Furthermore, NFs exhibited many benefits for wound healing, such as good mechanical attributes, flexibility, porosity, and drug capacity [11]. They could promote hemostasis, inhibit bacterial infections, and increase cell proliferation, aiding in the healing of wounds [12,13,14]. Natural remedies that possess anti-inflammatory, antioxidant, antibacterial, and pro-collagen formation are considered promising strategies for the acceleration of wound healing [15].

It has been reported that *Dragon’s blood* (DB) and the root extract of *Alkanna tinctoria* (AT) were used for wound healing [16,17]. DB has many medicinal properties, including wound healing, analgesic, antiulcer, antibacterial, antiviral, antihemorrhagic, anti-inflammatory, antioxidant, mutagenic, and cytotoxic effects [16]. The main elements of DB responsible for its wound-healing properties are proanthocyanidins, taspine, and lignans [17].

In addition, the root extracts of AT have been used for wound healing since ancient times and in the treatment of burns, macular eruptions, and infectious diseases, such as measles [18]. The major active constituents of AT root extract are the isohexenylnaphthazarins, known as alkannin and shikonin (A/S), which, along with their ester derivatives, may act as potent antimicrobial, anti-inflammatory, antioxidant, proliferative, and wound-healing agents [17,19]. AT wound-healing properties have also encouraged its use in traditional ointments and pastes for treating wounds [17]. 

Therefore, the purpose of this current study was to explore the potential synergistic effects of combining DB and AT to promote wound healing, particularly in diabetic conditions. To the best of our knowledge, no studies have assessed electrospun NFs loaded with both DB and AT and their ability in wound-healing applications. PVP polymer was utilized in an electrospinning technique to obtain NFs that co-loaded DB and AT. PVP is known for its biodegradability, biocompatibility, mucoadhesiveness, high loading capacity, and the ability to dissolve in water and organic solvents [20]. Standard tests, such as antibacterial and histological evaluations, were carried out to verify the fabrication process and appraise the biological capabilities of the wound dressing. Therefore, the antibacterial potential and wound-healing efficacy of the dressing NFs using in vitro and in vivo studies were investigated. 

## 2. Materials and Methods

### 2.1. Materials

PVP polymer with an average molecular weight of ~1,300,000, streptozotocin with Mwt ~265.22 g/mol, phosphate-buffered saline (PBS, pH 7.4), acetic acid, and absolute ethanol (absolute, ≥99.5%) were purchased from Sigma-Aldrich (St. Louis, MO, USA). DB powder was purchased from Maya Ethnobotanicals (Haarlem, The Netherland*s*)*,* while AT powder was obtained from a local store (Riyadh, Saudi Arabia). Milli Q Millipore (Billerica, MA, USA) was used to produce distilled water. All purchased materials were of the analytical grade.

### 2.2. Preparation of AT and DB Extracts

The ethanolic extract of AT root was prepared using a Soxhlet extractor. Briefly, 20 g of dried powder was added to 100 mL of 96% ethanol in the extraction tube. The apparatus was operated for 24 h to facilitate the extraction process. Subsequently, the liquid extract was filtered and subjected to drying in an oven at 50 °C. The resulting dried residue was carefully scraped and dissolved in 96% ethanol to attain the final volume of 2 mL, and then kept in a refrigerator (4 °C) [21]. 

For the extraction of DB, the dried powder was mixed with 100 mL of 80% ethanol and subjected to extraction using a Soxhlet apparatus for 4 h. Then, the mixture was filtrated and the filtrate was concentrated using a rotary evaporator (Heidolph, Schwabach, Germany). The concentrated extract was further subjected to freeze-drying using an Operon freeze-drier (Operon Co., Kimpo, Republic of Korea) to obtain the final product [16]. The resultant extracts were collected and stored at 4 °C.

### 2.3. Preparation of Extracts Co-Loaded Electrospun NFs

The electrospinning method was applied to create the PVP NFs based on the method of Tawfik et al. with some modifications [22]. A homogeneous polymer solution of 10% (*w*/*v*) PVP in ethanol was prepared at room temperature. Next, this polymer solution was mixed with either AT (0.5% *w*/*v*) or DB (0.5% *w*/*v*) extracts under stirring for 2 h. The electrospun NF preparation was carried out using a Spraybase^®^ electrospinner (Dublin, Ireland). Briefly, the plant extract/polymer solution was loaded into a 5 mL syringe equipped with a 0.9 mm needle. The spinneret-to-collector distance was set to 15 cm, and the flow rate was adjusted to 0.8 mL/hour. A constant voltage of 6.5 and 8 kV for AT and DB, respectively, was applied. The NFs of the combined plant extracts (i.e., DB and AT) were produced using the same parameters but at a constant voltage of 11 kV. Blank NFs were also produced using the same parameters without the addition of the plant extracts and with a constant voltage of 5.6 kV. The collector was covered with aluminum foil to collect and peel off the fibers easily after processing. All electrospinning processes were performed at room temperature and 40% relative humidity.

### 2.4. Scanning Electron Microscopy and Diameter Assessment 

The surface morphology and diameter of the electrospun NFs were examined using JEOL Scanning Electron Microscopy (SEM) (JSM-IT500HR, ASIA PTE. Ltd., Singapore). The samples (0.5 cm) were cut and mounted on carbon tape stubs, and coated with platinum utilizing the JEOL auto fine coater (JEC-3000FC, ASIA PTE. Ltd., Singapore). The prepared samples were then observed under the SEM. The average diameters of approximately 45 NFs were measured using the SEM-ImageJ software (National Institute of Health, Bethesda, MD, USA).

### 2.5. UV-Visible Spectrophotometry

The wavelengths of the maximum absorption for the extracts from AT, DB, and plain NFs were identified. Subsequently, these samples were serially diluted to form several concentrations. Utilizing a UV-visible spectrophotometer (Thermo Scientific GENESYS 10S UV-VIS, Madison, WI, USA), the absorbance values were measured, and a calibration curve was constructed to assess linearity. The standard solutions of AT and DB with concentrations ranging from 1.65 to 100.00 µg/mL were prepared. Calibration curves were constructed at 275 and 228 nm, corresponding to the maximum wavelengths for AT and DB*,* respectively.

### 2.6. Loading Capacity and Entrapment Efficiency of the Extract-Loaded NFs

The entrapment efficiency (EE%) and loading capacity (LC) of AT, DB, and their combination-loaded NFs were measured using UV spectrophotometry (Thermo Scientific GENESYS 10S UV-VIS, Madison, WI, USA). The certain weight (~4 mg) of the electrospun NFs was dissolved in a 10 mL solvent mixture of PBS (pH 7.4) and acetonitrile at (80:20) volumetric ratio and left at room temperature for 6 h to ensure complete dissolution. The samples were then filtered through 0.22 mm and detected by UV spectrophotometry at λ_max_ of 275 and 228 nm using standard curves for AT and DB*,* respectively, after dilution. Blank NFs were also measured using the same dilution and wavelengths to serve as the reference blank solution. All measurements were conducted in triplicate. The EE% and LC values were calculated using the following equations:

(1) $$EE\;\left(\%\right)=\frac{Amount\;of\;extract\;entrapped}{Total\;amount\;of\;extract}\times 100$$

(2) $$LC\;(µg/mg)=\frac{Amount\;of\;extract\;entrapped}{Actual\;amount\;of\;nanofiber}$$

### 2.7. X-ray Powder Diffraction (XRPD)

The crystallinity of the electrospun NF content was evaluated. XRD scans were acquired for the electrospun NFs using a RIGAKU diffractometer (RigaKu Corporation, Tokyo, Japan), equipped with a graphite crystal monochromator, automatic divergence slit, and automatic controller (PW/1710). Cu Kα radiation working at 40 KV and 40 mA (k = 1.54056 Å) was used as the target. The diffraction measures were achieved using continuous scan mode with a 2θ degree range from 3° to 60° at a scan speed of 1.00°/minute.

### 2.8. Water Solubility of the Electrospun NFs

The dissolving test of the blank NFs and plant extract-loaded NFs was studied. Approximately 4 mg of each NF were placed into 8 mL of pre-warmed PBS (pH 7.4) in Petri dishes. Experimental conditions were conducted in a thermostatic shaking incubator at room temperature. The dissolving property was recorded by continuous shooting mode. Each sample was tested in triplicate. 

### 2.9. In Vitro Extract Release 

The release profiles of AT and DB from electrospun NFs were studied by UV-Vis spectrometry. Briefly, the electrospun NF samples weighing 6–8 mg in a dialysis bag (12–14 KDa) were immersed in 40 mL of phosphate-buffered saline (PBS, pH = 7.4) containing tween 80 (0.5%) in a shaker water bath at 37 °C and a speed of 100 RPM in a dark room. At predefined time intervals (up to 360 min), 2 mL of the release medium was withdrawn. Simultaneously, an equal volume of the fresh PBS solution was replaced to preserve the sink conditions. The concentration of AT and DB in the release medium at each time point was measured at 275 and 228 nm using a UV-Vis spectrophotometer. All measurements were performed in triplicate to ensure reliability. 

### 2.10. Antibacterial Activity

The in vitro antibacterial activity of AT, DB, and DB-AT, and their incorporation into NFs was evaluated against Gram-positive *Staphylococcus aureus* (*S. aureus*) ATCC 29213 and Gram-negative *Pseudomonas aeruginosa (P. aeruginosa)* ATCC 27853 using the agar well diffusion method [23].

Firstly, the antibacterial susceptibility of AT, DB, and DB-AT extracts, and their loaded NFs was evaluated using the agar well diffusion method [21]. The microbial strains *S. aureus* and *P. aeruginosa* were grown in a nutrient broth culture medium for 24 h at 37 °C at 200 rpm in an orbital shaker. The bacterial inoculum (0.5 McFarland turbidity) was spread uniformly on a Mueller/Hinton agar separately using sterile cotton swabs. Then, 7 mm wells were formed aseptically on an agar plate using a sterile cork borer. Two concentrations of AT, DB, and DB-AT extracts (5 and 10 mg/mL) were added to the respective wells. DMSO (negative control) and standard antibiotics vancomycin and penicillin were used for positive controls for *S. aureus* and *P. aeruginosa*, respectively. 

Additionally, to evaluate the susceptibility of AT, DB, and their combined loaded NFs, a specific weight of each NF (2.5 mg/mL) was investigated. The plates were preserved at 4 °C for 1 h and then incubated aerobically for 24 h at 37 °C. The antibacterial activity was determined by measuring the average diameter of the inhibition zones. The minimum inhibitory concentration (MIC) of the NFs loaded with AT, DB, and DB-AT was determined using the agar well diffusion method against *S. aureus* and *P. aeruginosa* as described above. The suspensions of different weights of each NF (2.5, 1.25, 0.62, 0.31, and 0.15 mg/mL) were added to each well separately. The lowest weight, corresponding to the lowest concentration of the extract that exhibited an inhibition zone, was considered the MIC for each sample. 

### 2.11. In Vivo Wound Healing Study

Wistar rats (weighing ~250 g) were obtained from the College of Medicine, King Saud University, Riyadh, Saudi Arabia. The animals were housed in standard cages with each cage containing 4 rats at 22 ± 2 °C and relative humidity (50 ± 5%) with a 12 h light/dark cycle. The animals were given standard rodent chow and free access to water and the body weight and food intake were recorded. The animals were left to acclimatize to the animal house conditions for 1 week before the start of the experiments. They were handled humanely and gently before and during the entirety of the experiment according to the guidelines of the National Institute of Health, USA. The animal experiment in this study was carried out according to the Guidelines of the Research Ethics Committee of King Saud University College of Pharmacy, Riyadh, Saudi Arabia (Ref. No.: KSU-SE-22-74). 

#### 2.11.1. Experimental Design of Wound Healing Study

The streptozotocin-induced diabetic model in Wistar rats was employed for the in vivo assessment of diabetic foot wound closure. The rats were intraperitoneally injected with 60 mg/kg of streptozotocin (in pH 4.5 sodium citrate buffer, 0.1 mol/L). The blood samples were collected from the tail vein on days 3, 10, and 17 post-injection, and the plasma glucose concentration was measured using a blood glucose monitor. The rats exhibiting consecutive fasting blood glucose levels exceeding 250 mg/dL on both days 10 and 17 were classified as chronically diabetic and included in the study. Additional parameters, such as fasting morning plasma glucose levels, water consumption, and body weights, were assessed at weekly intervals. The rats with two consecutive fasting blood glucose levels higher than 250 mg/dL on both days 10 and 17 were considered chronically diabetic and included in the study [16,24].

Following anesthetic induction through the intraperitoneal injections of ketamine (50 mg/kg) and xylazine (5 mg/kg), the dorsal hair was shaved to create wounds. The skin was sterilized using a betadine solution. Next, a single circular full-thickness wound, utilizing a disposable 8 mm skin biopsy punch, was created. The rats were then divided into 10 groups, each including 5 rats, further divided into diabetic (5 groups) and non-diabetic (5 groups) subsets. The normal rat group received the blank NFs, while the control group received sterilized gauze. The treated groups were AT NFs, DB NFs, and DB-AT NFs. They were applied to wounds by putting them (5 × 5 mm^2^) in touch with the subcutaneous tissue. Every NF-covered injury was protected using a Tegaderm^TM^ and adhesive fabric tape to prevent bacterial contamination. The rats were returned to the housing and maintained with normal food and water [25]. Every day, the treatment protocol was followed, and the injury was covered with fresh NFs. The wound margins were observed and photographed on different days (0, 3, 7, and 14 days after surgical incision) to monitor the changes in the size of the wounds using an electronic digital caliper. The original area (day 0 area) was identified as the area identified by the trace obtained immediately after wounding. Wound closure is expressed as percentage closure relative to the original size and was calculated according to the following equation:

(3) $$Wound\;closure\;\left(\%\right)=\frac{Initial\;wound\;area-Nth\;day\;wound\;area}{Initial\;wound\;area}\times 100$$

#### 2.11.2. Histopathological Analysis 

On day 14, the rats were sacrificed humanely by increasing the concentration of CO_2_ until they became completely unresponsive, and then dorsal skin tissue was dissected. They were subjected to a histological analysis, and then the sacrificed rats were eliminated according to KSU laboratory protocol. For histopathology, the tissues were embedded in 10% phosphate-buffered formalin at 4 °C for 24 h. After fixation, the samples were dehydrated using ascending grades of alcohol, embedded in paraffin, and processed using a rotary microtome to prepare 5 μm thick paraffin sections. For staining, the sections were deparaffinized and stained with hematoxylin and eosin (H&E) and examined under a light microscope to detect the structural changes [17,25].

### 2.12. Data Analysis:

The data were expressed as mean ± standard deviation and analyzed using the two-tailed Student’s *t*-test to compare the mean duration of wound healing between the two groups and the percentage of wound healing per day. The analysis of variance (ANOVA) method was used to compare the mean duration of healing between different anatomical areas. A value of *p* < 0.05 was considered significant.

## 3. Results and Discussion

Natural substances like AT- and DB-loaded electrospun NFs possess the potential to improve wound healing, particularly in diabetic lesions. They could serve as effective anti-infection wound dressings. These herbal extracts can be regarded as a valuable alternative to conventional antibacterial agents. The obtained NFs incorporated with AT, DB, and their combination were investigated for their diameter and morphology to assess their suitability for healing applications. The electrospinning technique has proven efficacy in producing nanofibrous structures for tissue engineering purposes. Furthermore, AT and DB are known to exert diverse mechanisms that actively support the different stages of wound healing. In this work, NFs with extracts from AT and DB were created, and their ability to accelerate the healing of diabetic lesions was evaluated.

### 3.1. Surface Morphology and Diameter Characterization

**Figure 1:**
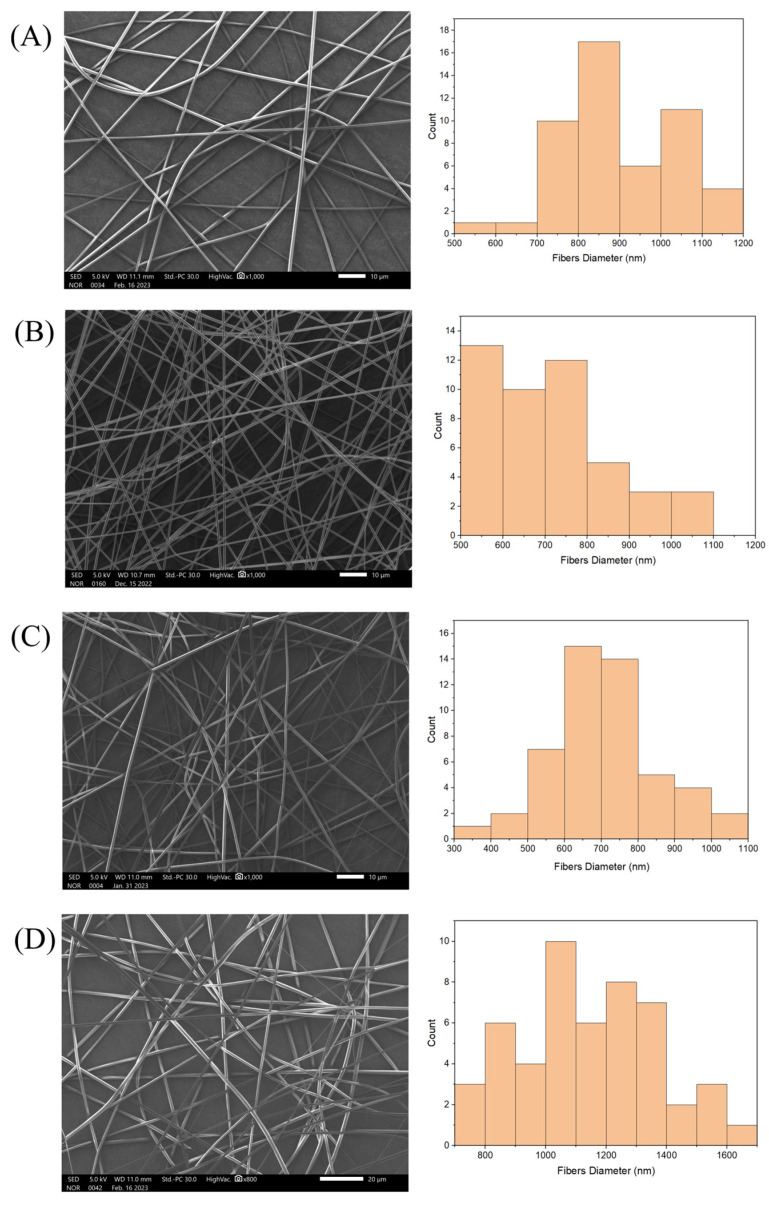
SEM images and diameter distributions of different electrospun NFs: (**A**) plain NFs, (**B**) AT NFs, (**C**) DB NFs, and (**D**) DB-AT NFs.

The surface morphology of the electrospun NFs exhibited a smooth surface which was bead- or pore-free. The electrospinning of 10% (*w*/*v*) PVP solution resulted in the uniform production of NFs with a smooth surface and an average diameter distribution of 893 ± 141 nm without any extract load (Figure 1A). The incorporation of 0.5% (*w*/*v*) of AT extract yielded thinner NFs with a uniform morphology and an average diameter of 688 ± 162 nm (Figure 1B). Similarly, SEM micrographs revealed that the loading of 0.5% (*w*/*v*) of the DB extract produced NFs with a uniform shape and a diameter distribution of 712 ± 137 nm (Figure 1C). This slight reduction in the diameter was due to the higher application of voltage necessary to adjust the spinning jet, which in turn decreased the diameter. Upon adding 0.5% (*w*/*v*) AT-BD into the PVP fibers, the diameter of the prepared NFs was increased to an average of 1147 ± 234 nm (Figure 1D). This increase was probably due to the increasing viscosity of the combination of the two extracts and the physical intermolecular interaction between the polymer and the extracts [15,26]. Overall, the obtained formulations showed a successful preparatory criterion, the lack of beads, and pore formation, which were consistent with other drug-loaded PVP NF studies [27,28,29].

### 3.2. Electrospun NF Solubility Study

**Figure 2:**
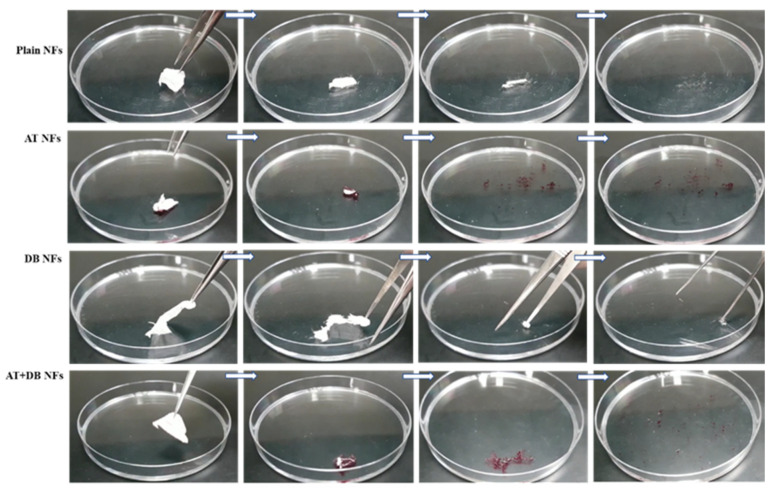
The solubility of the plain NFs and AT NFs showing an ultra-rapid dissolving (<2 s) compared to the DB NFs and DB-AT NFs (>30 s).

As depicted in Figure 2, four types of electrospun NFs were added to PBS (7.4). Both plain and AT NFs exhibited rapid dissolution in less than 2 s, whereas the DB NFs and DB-AT NFs were dissolved in more than 30 s despite using the hydrophilic polymer PVP. The obtained NFs initiated the dissolution process by transforming into a gel-like material, eventually dissolving completely. The extended dissolution duration observed in NFs containing DB might be due to the pronounced hydrophobic nature of DB as evident in Figure 2 since the extract exhibits a violet color. The quick solubility of the obtained NFs could be attributed to the employed polymer, i.e., PVP, the nanoscale porosity, and the large surface area of the electrospun NF matrix [30]. Additionally, this rapid dissolution of the electrospun NFs could contribute to the fast drug release rate and efficient wound-healing outcomes. This distinctive property of NFs may contribute to keeping the wound in a moist environment for efficient wound healing, which would enhance the therapeutic efficacy of the loaded extracts [31].

### 3.3. Loading Capacity and Entrapment Efficiency of the Extract-Loaded NFs

The extract-loaded electrospun NFs exhibited EE% values of 51.3% for the AT-NFs and 53.2% for the DB-NFs, respectively. In their combined forms, AT showed 42.2% for EE%, while the DB-NFs had 49.6% for EE%. The LC of the AT- and DB-loaded NFs were 64.1 and 70.4 µg/mg, respectively. Additionally, in their combined NFs, the amounts of AT and DB were 49.6 and 57.2 µg/mg, respectively. These low encapsulations of the extracts might be explained by their loss during the removal of the PVP-electrospun NF membrane from the aluminum foil and some fibers were deposited onto the walls of the instrument chamber [27]. Additionally, this low loading capacity could also indicate a lack of interaction between the polymer and the extract, which affected their integration and encapsulation within the fibers, which will need further investigation.

### 3.4. XRD Analysis

**Figure 3:**
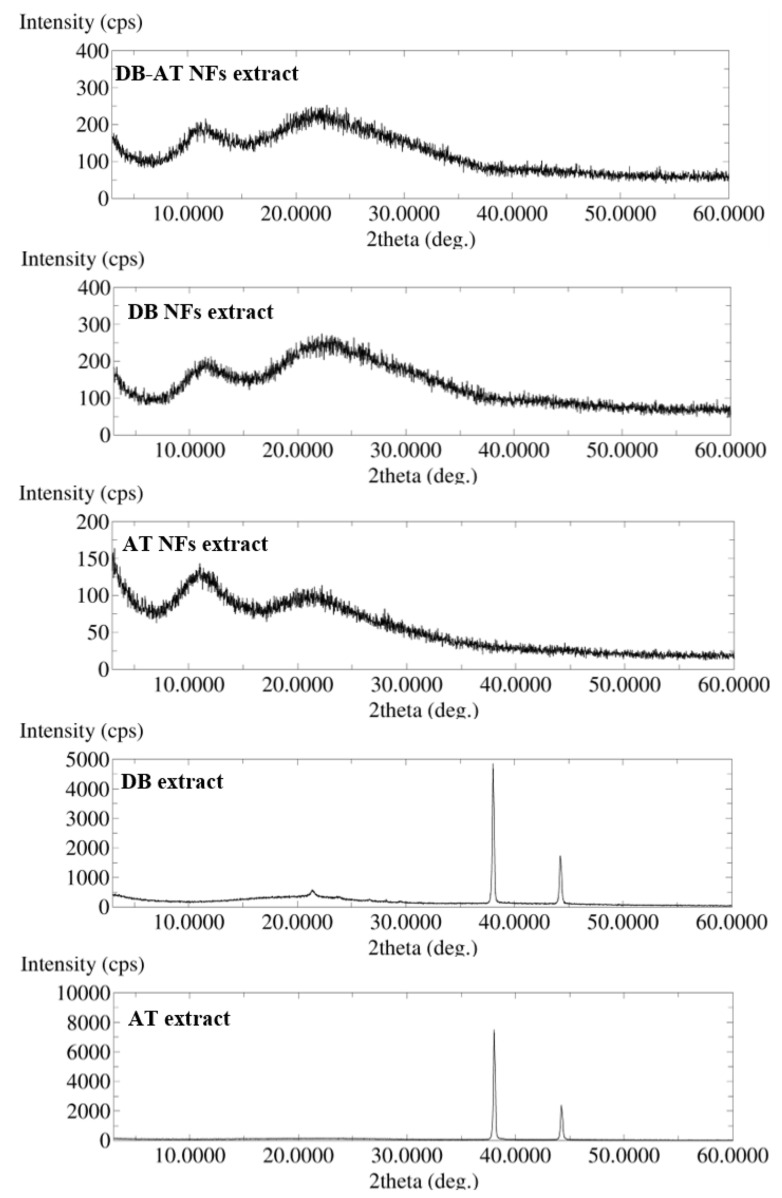
XRD spectra of the AT, DB, AT NF, DB NF, and DB-AT NF electrospinning process. The diffractograms show the distinctive peaks of AT and DB, which were lacking in the NF systems indicating their molecular dispersion.

The physical state of the AT, DB, AT-NFs, DB-NFs, and DB-AT NFs was studied using XRD, as shown in Figure 3. The crystalline structures of the two extracts were detected. The AT pattern displayed a sequence of prominent Bragg reflections at 2θ: 39° and 44°. Meanwhile, the DB extract exhibited strong Bragg reflection at 2θ: 21°, 38°, and 44°.

These intense characteristic peaks of the plant extracts were absent in the extract-loaded NFs, suggesting their molecular transformation owing to the electrospinning process as reported in the previous studies [28,30,32]. This molecular transformation may be attributed to the rapid solvent evaporation during the electrospinning, which prevented the molecules from organizing into the crystalline lattice and causing disordered arrangements in the NFs [33].

### 3.5. The In Vitro Release Study

**Figure 4:**
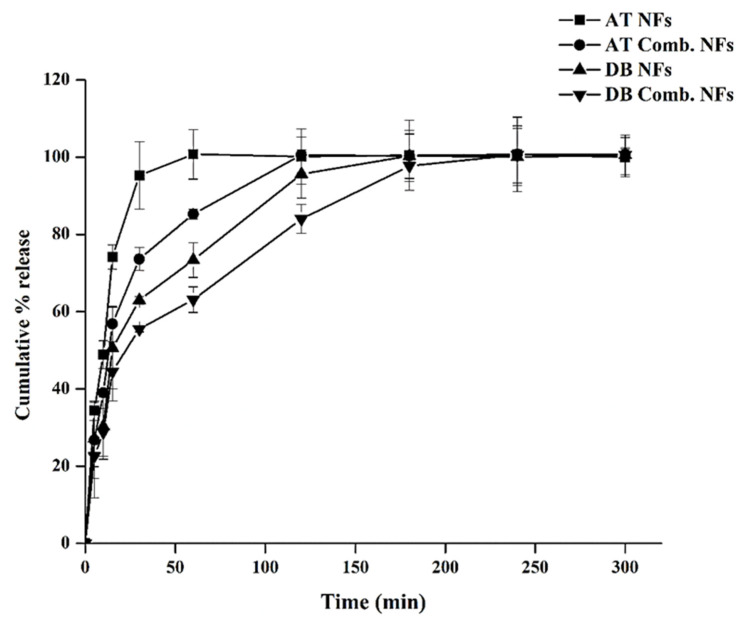
The in vitro release profile of the AT-, DB-, and their combined-loaded NFs, and the results are represented as mean ± SD (n = 3). The rapid release of AT compared to the DBNFs was observed.

The AT-, DB-, and their combined-loaded NFs exhibited a rapid initial burst release within the first 10 min for both the AT and DB NFs (Figure 4). This phenomenon was anticipated due to the hydrophilic nature of the PVP polymer, which facilitated the dissolution of the NFs and accelerated the release of the loaded drugs [27,28]. After 30 min, the release of AT NFs and DB NFs was approximately 70% and 62%, respectively, while the release of DB-AT from the NFs was approximately 59% for AT and 46 for DB. After 60 min, the release of the AT NFs and DB NFs reached the maximal level with about 100% and 73% for AT and DB, respectively, while the release of DB-AT from the NFs was about 84% for AT and 60% for DB. The release for all NFs (~100%) was detected after 180 min (Figure 4).

The enhanced contact area between the NFS and the dissolution medium is attributed to their high surface/volume ratio, thereby facilitating the rapid release of the drugs [29]. Moreover, the slightly slower release of DB from its NFs compared to AT may be attributed to the slower dissolving of these fibers.

### 3.6. Antibacterial Activity

**Figure 5:**
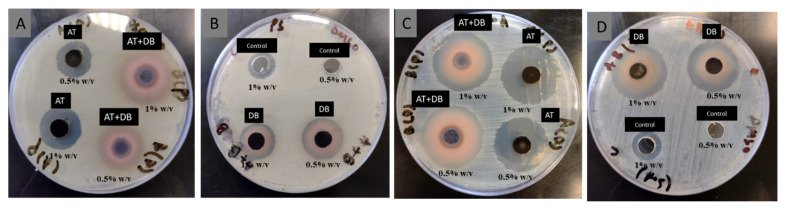
Antibacterial activity of AT, DB, and AT + DB extracts against *P. aeruginosa* (**A**,**B**) and *S. aureus* (**C**,**D**).

*S. aureus* and *P. aeruginosa* are the most prevalent pathogens resulting in infected diabetic wounds [34]. The antibacterial activity of the DB, AT, DB-AT, and loaded-NFs against *S. aureus* and *P. aeruginosa* using the agar well diffusion method was evaluated. Two concentrations (5 and 10 mg/mL) of AT, DB, and the AT-DB mix were tested. Penicillin was used as a positive control for *P. aeruginosa* and vancomycin for *S. aureus* bacteria. The zone of growth inhibition for each extract was measured (Figure 5) and (Table 1). The extract of DB, AT, and DB-AT showed significant antibacterial activity at the concentrations of 0.5 and 1.0% *w*/*v* against *P. aeruginosa* and *S. aureus.*

**Figure 6:**
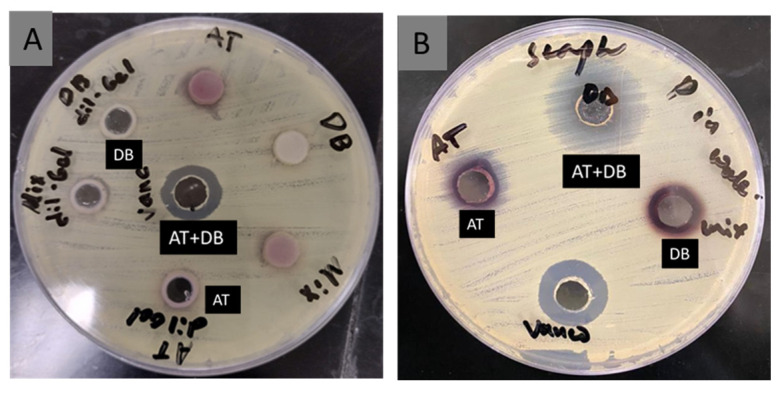
Antibacterial activity of AT, DB, and AT-DB NFs using 0.5% *w*/*v* of NF samples against (**A**) *P. aeruginosa* and (**B**) *S. aureus*.

Furthermore, the investigated NFs showed antibacterial activity against *P. aeruginosa* and *S. aureus* (Figure 6) at the weight of 0.5% *w*/*v* from the NFs. However, *S. aureus* showed a greater susceptibility to the examined NFs compared to *P. aeruginosa.*

**Figure 7:**
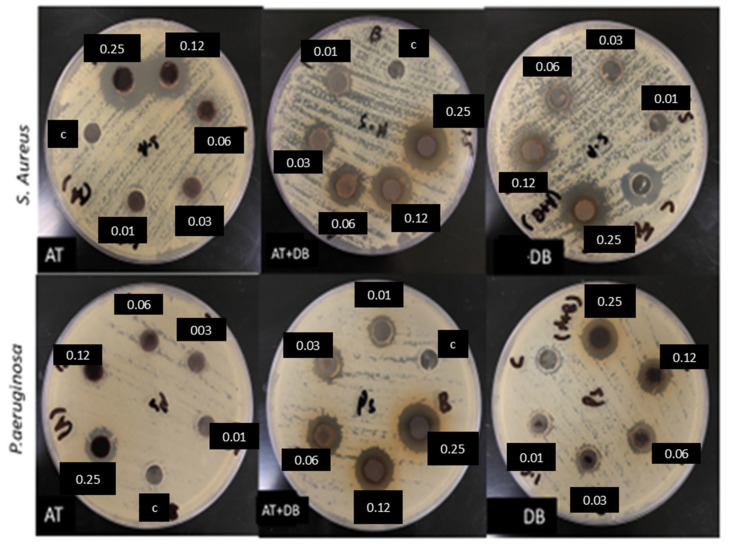
MIC of AT, DB, and DB-AT-loaded NFs at 0.25, 0.12, 0.06, 0.03, and 0.01% *w*/*v*; control (c) is blank NFs.

MIC of the DB-, AT-, and DB-AT-loaded NFs was also tested through agar diffusion assay against *S. aureus* and *P. aeruginosa* (Table 2). The starting concentration of the NFs was 0.25% *w*/*v*, followed by double-fold serial dilution, which was then carried out across the plate. The lowest concentration was 0. 01% *w*/*v*. As shown in Figure 7, the MIC values against *P. aeruginosa* were determined as follows: 0.25% *w*/*v* for the AT NFs, 0.06% *w*/*v* for the DB NFs, and 0.03% *w*/*v* for the DB-AT NFs, which corresponded to concentrations of 80 µg/mg for the AT NFs and 22 µg/mg for the DB NFs, while the DB-AT NFs had 10 µg/mg of DB and 8.5 µg/mg of AT.

In the case of *S. aureus,* the MIC values were 0.12% *w*/*v*, 0.06% *w*/*v,* and 0.01% *w*/*v* for the AT NFs, DB NFs, and DB-AT NFs, respectively. These values corresponded to concentrations of 160 µg/mg for the AT NFs, and 44 µg/mg for the DB NFs, while DB-AT had 10 µg/mg for DB and 8.5 µg/mg for AT. The NFs showed significant activity against both bacterial strains. The results indicated that the DB-AT NFs demonstrated superior antibacterial activity against *S. aureus* at the lowest concentration of 0.01% *w*/*v*.

The antibacterial activity of AT in *S. aureus* is consistent with the findings of a previous study conducted by the Khan group [35]. The antibacterial activity of DB could be attributed to the presence of proanthocyanidin, which can prevent bacterial infections effectively [36].

### 3.7. In Vivo Wound Healing

**Figure 8:**
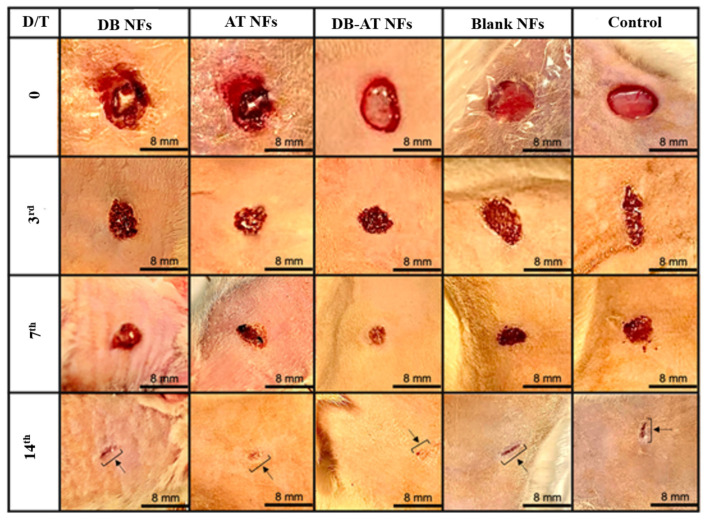
In vivo wound healing on diabetic rats. The variations in average values between test and controls have been determined and expressed as mean ± SD. DB-NFs: *Dragon’s blood*-loaded nanofibers: AT-NFs: *Alkanna tinctoria*-loaded nanofibers; DB-AT-NFs: *dragon blood*-loaded nanofibers; DB-NFs: *dragon blood* and *Alkanna tinctoria* co-loaded nanofibers; blank NFs: blank nanofibers; positive control: sterile gauze.

**Figure 9:**
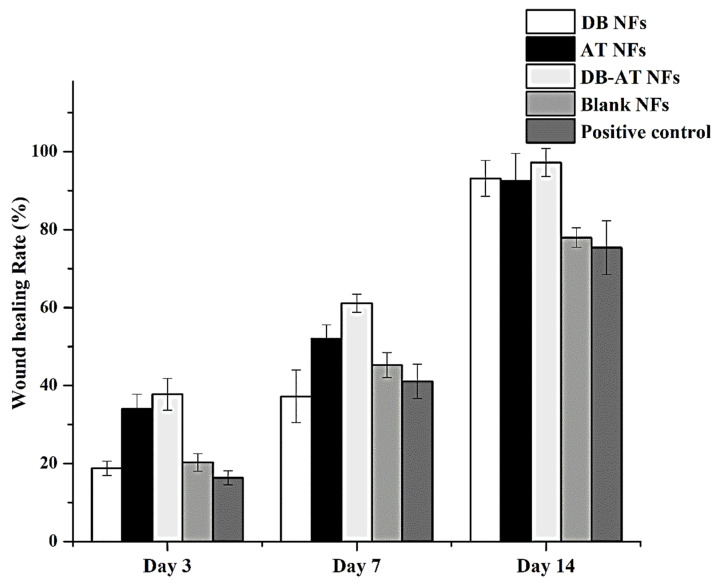
In vivo wound-healing rate of diabetic rats at 3, 7, and 14 days for different experimental groups.

This study aimed to evaluate the ability of electrospun NFs with high porosity to mimic the biologically and physically active extracellular structures. This behavior facilitates wound healing and tissue regeneration. The porosity of NFs supports nutrient exchange, ensuring the gas/liquid exchange of tissue cells and preventing excessive drying and dehydrating of the wound [8,9]. The unique structure of NFs is designed to enhance hemostasis with antibacterial efficacy, producing efficient wound healing [8]. The permeability of NFs contributes to a perfect microenvironment for cellular activity, helping effective tissue regeneration after wound dressing [10]. To explore if the extract-loaded NFs containing DB, AT, or both could help diabetic wound healing, the streptozotocin-induced wound model was used because it is closely similar to the pathological characteristics of chronic wounds, as has been reported in several publications [37]. Diabetic wounds cannot be healed easily because inflammatory cells are always present in the injured areas [38]. After the induction of wounds in diabetic Wistar rats, the wound sizes and healing rates were evaluated following treatment with the DB NFs, AT NFs, DB-AT NFs, blank NFs, or gauze (positive control). The images of wound healing and rates are illustrated in Figure 8 and Figure 9, respectively. Figure 8 displays the wound areas in diabetic rats that were treated with the DB NFs, AT NFs, DB-AT NFs, blank NFs, and gauze (positive control). Figure 9 displays the wound healing of the rat on the 3rd day, revealing healing rates of 18.79 ± 1.8%, 33.99 ± 3.8%, 37.75 ± 4.1%, 20.30 ± 2.26%, and 16.36 ± 1.8% for the DB NFs, AT NFs, DB-AT NFs, blank NFs, and positive control, respectively. Notably, the DB-AT NFs exhibited the highest healing rate among all treatments. By the 7th day, the healing rates increased to 37.23 ± 6.7%, 52.07 ± 3.5%, 61.14 ± 2.3%, 45.23 ± 3.17%, and 41.09 ± 4.42% for the DB NFs, AT NFs, DB-AT NFs, blank NFs, and positive control, respectively. The DB-AT NFs maintained the highest healing rates. After two weeks of treatments, the healing rates were 93.15 ± 4.6%, 92.51 ± 7.0%, 97.20 ± 3.6%, 77.94 ± 2.50%, and 75.39 ± 6.89% for the DB NFs, AT NFs, DB-AT NFs, blank NFs, and positive control, respectively. The black arrows showed that all wounds in each group were almost completely healed by day 14 (Figure 12).

Thus, the DB-AT NFs continued to demonstrate superior healing rates compared to the other groups of treatments. Overall, the treatment with the DB-AT NFs resulted in improved healing rates and wound closure.

The enhanced healing observed with the DB-AT NFs may be attributed to the synergistic effects of both components, which promote cell proliferation, angiogenesis, and anti-inflammatory and antibacterial effects [16,17]. Therefore, the DB-AT NFs could be considered a multifunctional wound dressing. Additionally, the high porosity of the electrospun NFs enables efficient oxygen permeation, which might have contributed to improved wound healing [39].

**Figure 10:**
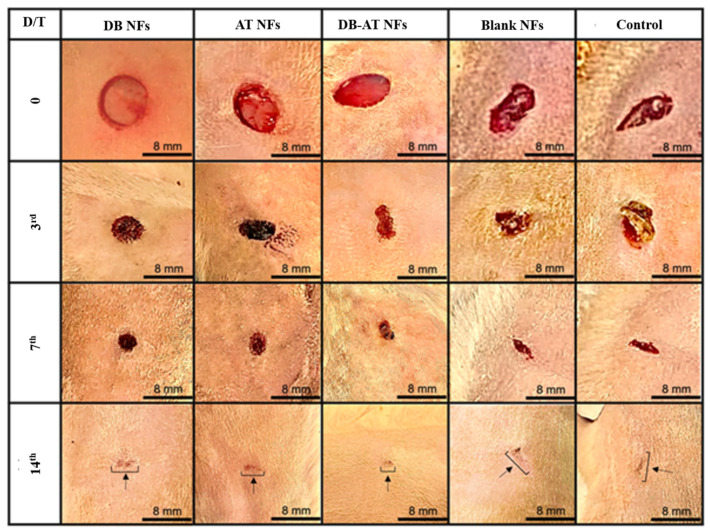
In vivo wound healing on control rats. The variations in average values between test and controls have been determined and expressed as mean ± SD. DB-NFs: *dragon blood*-loaded nanofibers: AT-NFs: *Alkanna tinctoria*-loaded nanofibers; DB-AT-NFs: *dragon blood*-loaded nanofibers; DB-NFs: *dragon blood* and *Alkanna tinctoria* co-loaded nanofibers; blank NFs: blank nanofibers; positive control: sterile gauze.

Furthermore, Figure 10 depicts the images of the wound areas in control rats (non-diabetic) treated with the DB NFs, AT NFs, DB-AT NFs, blank NFs, or gauze (positive control). On the 3rd day, the wound-healing rates observed were 30.04 ± 9.4%, 39.23 ± 7.1%, 53.82 ± 11.57%, 21.03 ± 3.8%, and 24.9 ± 9.4% for the DB NFs, AT NFs, DB-AT NFs, blank NFs, and gauze (positive control), respectively. By the 7th day, the rates of wound healing were increased to 69.71 ± 10.1%, 74.55 ± 9.7%, 76.69 ± 10.62%, 53.69 ± 2.5%, and 67.17 ± 7.6% for the DB NFs, AT NFs, DB-AT NFs, blank NFs, and gauze (positive control), respectively. Compared with the other groups of therapy, the DB-AT NFs showed superiority in healing rates as detected in diabetic rats. After 14 days, the wound recovery rates were 94.57 ± 3.4%, 94.68 ± 1.11%, 98.02 ± 3.53%, 86.31 ± 3.9%, and 91.63 ± 2.8% for the DB NFs, AT NFs, DB-AT NFs, blank NFs, and gauze (positive control), respectively. The DB-AT NFs continued to show the best healing rates in control rat models with the 14-day treatment.

It is important to note that the healing rate in the control rats is better than in the diabetic rats. This behavior of diabetes is due to physiological complications such as impaired blood flow, a weakened immune system, and increased oxidative stress, which lead to delayed healing [38]. The results suggest that the wound-healing rate was in the order of the DB-AT NFs > DB NFs > AT NFs > positive control > blank NFs, indicating that the DB-AT NFs are more effective for diabetic wound-healing.

**Figure 11:**
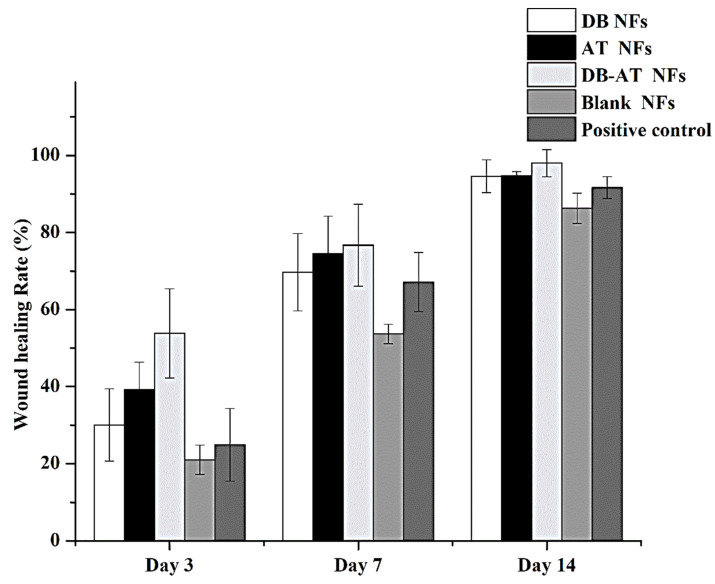
In vivo wound-healing rate of normal rats at 3, 7, and 14 days for different experimental groups.

**Figure 12:**
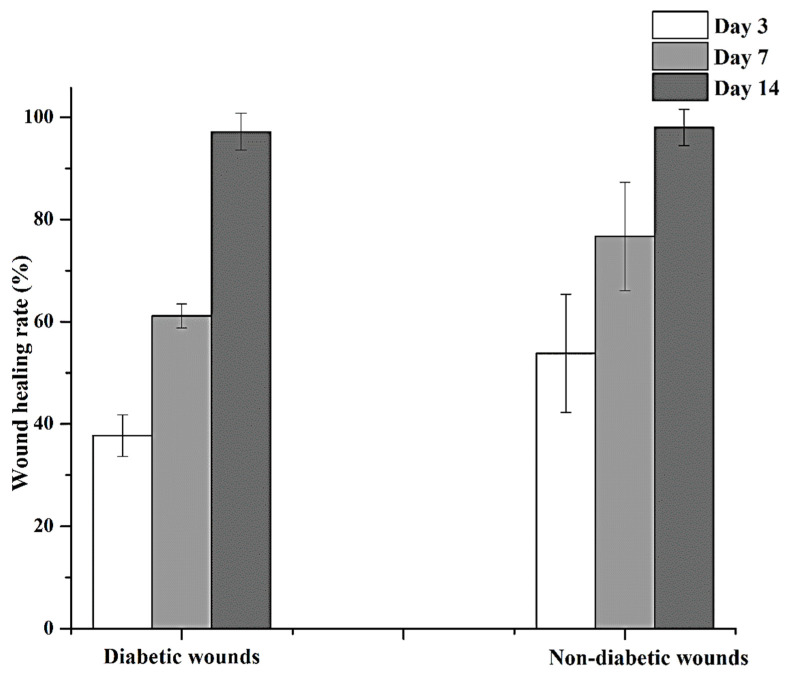
In vivo wound-healing rate using DB-AT NFs at 3, 7, and 14 days.

According to the obtained results, the non-diabetic wounds treated with the DB-AT NFs healed faster than the diabetic wounds on days 3 and 7 (Figure 11). The remarkable effect of the DB-AT NFs was also found in in vivo studies involving diabetic-treated groups. The black arrows indicate that all wounds in each group were almost completely healed by day 14 (Figure 12). Interestingly, the highest wound healing with observed the groups treated with the AT-DN NFs (~98%) as shown in Figure 12. These results suggest that combining the extracts of DB- and AT-loaded NFs presents a promising avenue to treat wounds that are hard to heal. The enhanced wound healing observed in the AT-treated group can be attributed to the presence of iso-hexenylnaphthazarins, specifically alkannin and shikonin (A/S), deoxy shikonin, and acetyl shikonin [18,19]. Similarly, the wound-healing activity of the DB extract is due to its rich content of proanthocyanidin and catechin, alkaloid taspine, and a high percentage of polyphenolic compounds [16]. These components significantly contribute to anti-inflammatory, antibacterial, and antioxidant effects, which can reduce swelling and create a more favorable environment for healing. This decrease in inflammation causes the surrounding tissue to swell less, which lessens tissue damage and speeds up the healing process. Moreover, they could stimulate the proliferation and migration of fibroblasts, the formation of collagen, and epithelium regeneration, accelerating the proliferation and remodeling phases of the healing process [16]. Furthermore, these constituents could accelerate angiogenesis, tissue regeneration, and fibroblast proliferation by efficaciously impeding the nitric oxide production elicited by lipopolysaccharide [40]. A significant enhancement in wound healing was detected on the 3rd day, which may be due to the proliferative and anti-inflammatory effects of A/S in acute non-contaminated full-thickness skin defects [17]. In addition, these formulations can modulate the proliferative and inflammatory stages of wound healing [18,41]. The above-mentioned activities of the AT- and DB-containing nanofibers may be the mechanisms underlying the advantages in wound healing that have been observed.

Furthermore, Kheiri et al. have shown that using the AT extract ointment on the donor site after the removal of the skin graft has resulted in a significant reduction in the wound score when compared to the placebo group [19]. An in vivo study by Karayannopoulou et al. discovered that the combined alkannin and shikonin incorporated ointment exhibited good healing when applied to full-thickness burns in dogs. The injured areas’ perfusion and contraction rates were much higher than those of the group irrigated with Ringer lactate [18]. Irani et al. [14] and Namjoyan et al. [16] demonstrated that DB shows great wound-healing activity in rat wound models.

### 3.8. Histopathological Examination

**Figure 13:**
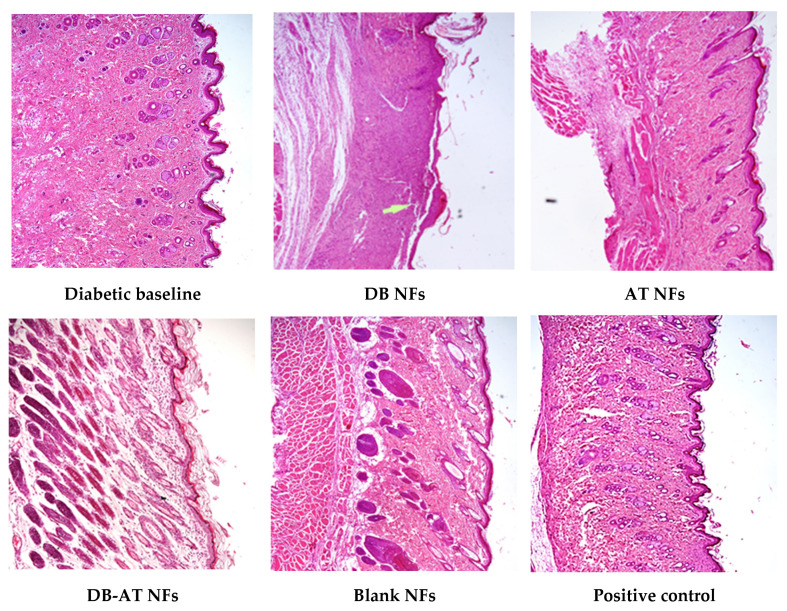
H&E staining images of the wound tissue on the 14th day of the diabetic rats’ groups.

**Figure 14:**
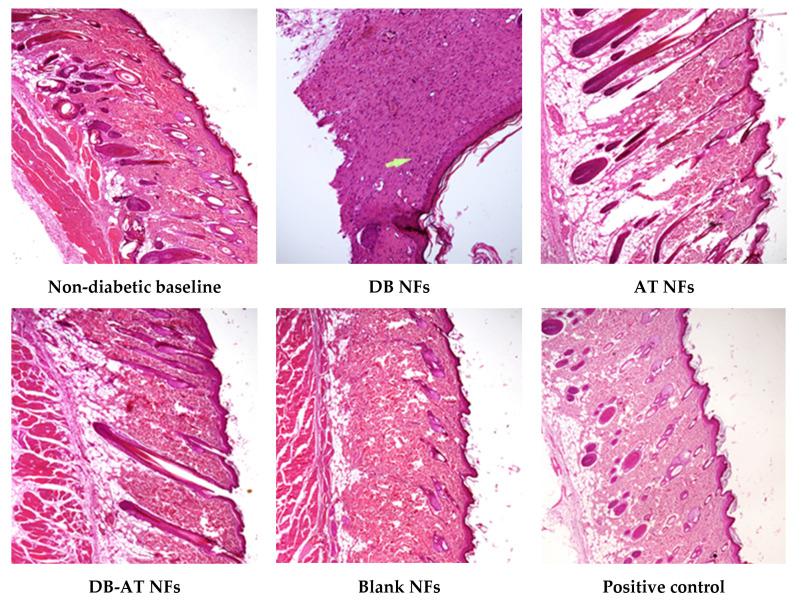
H&E staining images of the wound tissue on the 14th day of the non-diabetic rats’ groups.

H&E staining was used to evaluate the progression of wound healing at day 14 in both the diabetes (Figure 13) and non-diabetic (Figure 14) groups. The untreated groups showed a complete skin structure with dermis and epidermis. After treatment, notable differences in re-epithelialization and granulation tissue were observed (As shown by white arrows). In the positive control group that received gauze, the wounds appeared pink with a reduced size by day 14. Histological examination revealed a thin but complete epidermis with some rete ridges, suggesting the formation of a neo-epidermal layer. Additionally, minimal granulation and scar tissue were observed, indicating a more favorable healing process.

All groups treated with the NFs showed an immature regeneration of the epidermis and dermis layers, with the presence of neo immature blood vessels and skin appendages. The groups treated with the DB NFs displayed signs of delayed wound healing. These signs included the presence of epidermal ulcers. Additionally, a thin layer of neo-epidermal with no rete ridges, a smaller granulation tissue (2 × 1 mm) (as shown by white arrows), and a minor amount of scar tissue were detected. Additionally, the group treated with the AT NFs showed a pink appearance on day 14. Histologically, the signs of wound healing, like integrated neo-epidermis and the formation of rete ridges with minimal scar tissue, were exhibited. Moreover, the group treated with the DB-AT NFs displayed more pronounced signs of wound healing, both macroscopically and histologically. This group demonstrated a clear indicator of early wound healing with early neo-epidermal regeneration, maturation, and rete ridge formation, signifying complete wound healing. The granulation tissue and scar were minimal, further underscoring the advanced healing observed in this group. Moreover, the group treated with the DB-AT NFs showed a more pronounced regeneration and maturation of the epidermis and dermis layers.

Remarkably, the treated group with the DB-AT NFs showed clear indications of wound healing, with integrated epidermis and dermis tissue with skin appendages and capillary hyperproliferation signifying complete wound healing. The DB-AT NFs also demonstrated enhanced wound healing, which could provide greater therapeutic efficacy.

The present study established promising findings in the fabrication of the DB-AT NFs for wound healing. However, further experiments are required to ensure the safety and efficacy of these formulations, providing a deeper understanding of their impact. This highlights the need for preclinical studies on various animal models to determine optimal dosing and therapeutic outcomes. Additionally, stability studies should be conducted to evaluate storage conditions, ensuring they meet clinical standards. Complete safety and toxicity studies are also essential to confirm that these NFs are not only effective but also safe for clinical use.

## 4. Conclusions

PVP-electrospun NFs were successfully developed comprising two effective herbal extracts of DB, AT, and their combination. They were specifically designed to accelerate wound healing in diabetic foot applications. The obtained electrospun NFs demonstrated diameters in the range of 688 to 1147 nm with a desirable morphology, and loaded large amounts of AT and DB. Additionally, these NFs displayed high liquid solubility due to the hydrophilic nature of PVP, resulting in a rapid-release profile for all extracts. The rapid-release profile was obtained in the first hour with the initial burst release reaching 100% for AT and 73% for DB, while the release of co-loaded DB-AT from the NFs was about 84% for AT and 60% for DB. Afterward, a more gradual release pattern was observed with nearly 100% of all extracts from the NFs at 180 min. The DB-NFs and DB-AT NFs showed effective antibacterial activity against *P. aeruginosa* and *S. aureus*, resulting in reduced MIC values. Therefore, these NFs could play a vital role in wound-healing promotion. Particulary, in vivo, the study revealed that the created NFs facilitated the reduction in wound area and promoted fast healing, with the DB-AT-loaded NFs presenting a promising system for addressing wound healing in a diabetic rat model.

## Figures and Tables

**Table 1:** Zone of inhibition values for the extracts of AT, DB, and the AT-DB mix at the concentrations of 0.5 and 1.0 *w*/*v* against *S. aureus* and *P. aeruginosa*.

Microorganism	Concentration (*w*/*v*%)	Zone of Inhibition (mm)		
		DB	AT	DB-AT Mix
*S. aureus*	1.0	21	27	29
*S. aureus*	0.5	17	21	25
*P. aeruginosa*	1.0	20	19	22
*P. aeruginosa*	0.5	17	17	19

**Table 2:** MIC of AT-, DB-, and AT-DB mix-loaded NFs against *S. aureus* and *P. aeruginosa*.

Microorganism	MIC (%*w*/*v*)		
	DB	AT	DB-AT Mix
*S. aureus*	0.06	0.12	0.01
*P. aeruginosa*	0.06	0.25	0.03

## Data Availability

The authors confirm that the data supporting the findings of this study are available within the article.
